# Impact of maternal dTpa vaccination on the incidence of pertussis in young infants

**DOI:** 10.1371/journal.pone.0228022

**Published:** 2020-01-28

**Authors:** Frederico Friedrich, Maria Clara Valadão, Marcos Brum, Talitha Comaru, Paulo Márcio Pitrez, Marcus Herbert Jones, Leonardo A. Pinto, Marcelo C. Scotta

**Affiliations:** Centro Infant, Department of Pediatrics, School of Medicine, Pontifícia Universidade Católica do Rio Grande do Sul (PUCRS), Porto Alegre, Brazil; Universidad Nacional de la Plata, ARGENTINA

## Abstract

**Introduction:**

Pertussis is an important public health problem worldwide, especially in infants. An increase in the incidence in many countries occurred after 2010, including Brazil. In 2013, dTpa vaccine was introduced in the Brazil national immunization schedule of pregnant women. The objective of this study was to evaluate the national trends in the incidence of pertussis in Brazil in children under 1 year old, and the impact of the introduction of dTpa vaccine during pregnancy.

**Methods:**

The incidence of hospitalizations and non-hospitalized confirmed cases of pertussis in neonates (< 1 month age) and young infants (1 month—< 1 year age) were analyzed, comparing the incidence in pre maternal vaccination (2011–2013) with the post-vaccination (2015–2017). We used non-respiratory hospitalizations as comparison, during the same period. A database of the Brazilian Ministry of Health (DATASUS) was used to analyze cases from 2007 to 2017 and the subsets of 2011–2013 and 2015–2017, after Pertussis resurgence. The vaccination data was accessed through the link of the Information System of the National Immunization Program (pni.datasus.gov.br).

**Results:**

Between 2007 and 2017, 17,818 children under one year of age were hospitalized due to pertussis in Brazil. In the pre maternal vaccination period 2011–2013, the mean annual incidence of non-hospitalized confirmed cases of pertussis in children under 1 month was 722.2 / 100,000 and in the period of 2015–2017 the average was 377.3 / 100,000, representing a decrease of 47.7% [IRR 0.52 (0.46–0.59)]. At those periods of time, the average incidence per year for children of one month—< 1 year aged was 64.9 / 100,000 (2011–2013) and 29.3 / 100,000 (2015–2017) [IRR 0.45 (CI 0.29–0.69)].

**Conclusion:**

Vaccination of pregnant woman coincides with the reduction in the number of cases of pertussis in children under 1 month of age from 2015. Immunization of pregnant woman seems to have an important impact on the prevention of the disease in young infants who have not yet received their own pertussis vaccine.

## 1. Introduction

Considering the vaccine preventable diseases, pertussis is among the most prevalent bacterial diseases in several countries, including Brazil [1]. The World Health Organization (WHO) estimates 151,074 cases occurring, with 89,000 deaths recorded annually worldwide [2]. The disease is highly contagious and is caused by the bacterium *Bordetella pertussis*. The disease is characterized by severe cough, although young infants may only present episodes of apnea and/or cyanosis. It affects all age groups, being more severe in those younger than six months [3].

In the pre-vaccination era, the incidence of the disease in the United States of America reached 150 cases per 100,000 inhabitants per year. After the introduction of the vaccine in the 1940s there was a gradual decrease to 1/100,000 in the 1990s [4]. Since 2010–2012, there was an increase in the incidence of pertussis. In California, USA, it reached 138.4/100,000 inhabitants/year [5]. Despite widespread vaccination coverage, several regions of the world have observed this resurgence in the last decade. In 2014, the USA notified 28,660 cases [6].

The Brazilian National Immunization Program provides the whole-cell vaccine associated with diphtheria and tetanus (Triple Bacterial Vaccine—DTP) since its introduction in 1973, in three doses during the first year of age and other two boosters, one at 15 months and another at 4 years. This intervention led to the drop of 40,000 annual cases to 15,000 cases reported in 1990, with an incidence of 10.6/100,000 [7]. When vaccine coverage approached 95–100% in the years 1998–2000, the incidence decreased to 0.9/100,000, reaching 0.32/100,000 in 2010, with only 503 reported cases countrywide [8]. In Brazil, the number of confirmed cases has gradually changed with a progressive increase after 2011 [9]. It has been hypothesized that the increase in the incidence results from a combination of increased detection capacity through molecular biology techniques and possibly genetic mutations in *B*. *Pertussis* [10,11].

Immunization with triple bacterial vaccine acellular (dTpa) in pregnant women stimulates the production of antibodies against pertussis, which cross the placenta leading to the direct protection of the young infant and consists of a strategy recently introduced in several countries aiming to reduce the impact of the disease resurgence in young infants [12,13].

The diphtheria, tetanus and pertussis adsorbed vaccine (acellular pertussis) was introduced in November 2013 in the National Vaccination Calendar of the pregnant woman, with the goal of reducing the incidence and mortality due to pertussis in newborns and infants. Since then, in the year of its inclusion, the program reached a coverage of 9.3% of pregnant women in 2014, with a rise in the coverage in 2015, 2016 and 2017 to 44.9%, 33.8% and 42.1%, respectively. In infants, vaccination coverage was 94.8% in 2014 [14].

The objective of this study was to evaluate the national trends in the incidence of hospitalizations and non-hospitalized confirmed cases of pertussis in children under 1 month of age and those aged from 1 month to 1 year in Brazil; and the impact of the introduction of the maternal dTpa vaccine in these groups.

## 2. Materials and methods

Data from hospitalizations and notification of non-hospitalized confirmed cases of pertussis were obtained from the DATASUS database (http://datasus.saude.gov.br/) for the period of 2007–2017 and the subsets of 2011–2013 and 2015–2017 were analyzed after Pertussis resurgence [15]. The identification and notification of the cases is based on three criteria; Laboratory criterion: all suspected cases of pertussis with Bordetella pertussis isolation. Epidemiological criterion: Any suspected case that had contact with a case confirmed as pertussis by laboratory criteria, from the onset of the catarrhal period to three weeks after the onset of the paroxysmal period of the disease (period of transmissibility). Clinical criteria: Any suspected case in wich blood cell count shows leukocytosis (above 20,000 leukocytes / mm^3^) and absolute lymphocytosis (above 10,000 lymphocytes / mm^3^), provided that the following conditions are met: negative or unperformed culture results; no epidemiological bond and non-confirmation of another etiology [16].

The non-hospitalized confirmed cases were obtained through the links *Informações de Saúde* (Health Information) (DATASUS TABNET)–*Epidemiologicas e Morbidade* (Epidemiological and Morbidity)–*Doenças Notificadas* (Notification Diseases)—Pertussis, and finally being selected cases confirmed by age group and year of the first symptom. For the hospitalization data, the *Informações de Saúde* (Health Information) (TABNET) links–*Epidemiologicas e Morbidade* (Epidemiological and Morbidity)–*Morbidade Hospitalar* (Hospital Morbidity), *Lista de Morbidade* (Morbidity List) ICD-10 (Pertussis—A37.0) were used for the period 2007–2017, with the age group of < 1 year old of both sexes. Data from hospitalization in children under 1 year of age is not available by months (only years). Further information about DATASUS is described elsewhere [17,18].

To calculate the incidence of hospitalizations and non-hospitalized confirmed cases, we used the following formula: total number of hospitalizations or non-hospitalized confirmed cases / population number by age (per year and place [Brazil-IBGE]) x 100,000 inhabitants). To calculate the incidence of hospitalizations in children < 1 month of age, the following formula was used: total number non-hospitalized confirmed cases of pertussis in infants < 1 month of age / number of infants < 1 year of age divided by twelve (per year and local) IBGE ]) x 100,000 inhabitants) [19]. To calculate this, we divide by twelve the number of the children population under 1 year of age because there was no available data of population in months.

Non-respiratory hospitalizations were used as comparison (all ICD 10, excluding chapter X: J00 to J99), during the same period. To access data related to the vaccination of pregnant women, the Information System of the National Immunization Program (SI-PNI) was accessed [20] To assess the effect of inclusion of dTpa vaccine for pregnant women on the incidence of pertussis cases after the pre maternal vaccination period, the absolute reduction (pre-vaccination—post vaccination) and relative simple (pre-vaccination—post vaccination / pre vaccination) was calculated by analyzing the subsets 2011–2013 (pre) and 2015–2017 (post). The year 2014 was considered as transition due to the occurrence of both peak incidence and the introduction of the maternal vaccine dose. Main outcome were incidence of non-hospitalized confirmed cases due to Pertussis in patients aged less than 1 month, which is the cut-off of age available from DATASUS in children that are too young for vaccination. Secondary outcome was the incidence of non-hospitalized confirmed cases to Pertussis in patients aged 1 month—< 1 year. To calculate this, we divided by twelve the number of the population of children under one year of age and multiplied by eleven. To calculate the difference in incidence rates between the pre and post vaccine periods, incidence rate ratio was used to assess statistical significance, considering a 95% confidence interval.

To ensure quality, two independent authors reviewed all data. This study does not contains personal or individual data, so it was considered exempt from evaluation by the Research Ethics Committee of the Pontifical Catholic University of Rio Grande do Sul.

## 3. Results

From January 2007 to December 2017, there were 1,952,443 hospitalizations for respiratory diseases in children under 1 year of age in Brazil, and pertussis was responsible for 0.91% (17,818) of these cases. An increase in the incidence of hospitalizations due to Pertussis occurred after 2010, with the highest number occurring in 2014, reaching 199.8 / 100,000 / year among children under 1 year of age. In the pre maternal vaccination period 2011–2013, the average annual incidence of pertussis hospitalizations in children under 1 year old was 98.3 / 100,000 and in the period of 2015–2017 the average was 65.9 / 100,000, representing a decrease of 32.9% [IRR 0.67 (CI 0.49–0.91)]. Hospitalizations due to non-respiratory diseases increased significantly over the same period (Table 1).

**Table 1:** Hospitalizations for pertussis and non-respiratory diseases from 2007 to 2017 in children under 1 year of age in Brazil.

Year	Hospitalizations by pertussis (Incidence per 100.000 children)	Hospitalizations for non-respiratory diseases (Incidence per 100.000 children)
2007	719 (26.8)	384.255 (14347.96)
2008	836 (32.2)	365.350 (14087.66)
2009	604 (24.0)	371.043 (14722.28)
2010	352 (14.8)	381.933 (16057.06)
2011	1.061 (44.7)	377.257 (15905.21)
2012	2.588 (112.2)	384.768 (16686.14)
2013	3.080 (138.1)	391.366 (17547.03)
2014	4.333 (199.8)	403.803 (18616.42)
2015	2.184 (102.0)	417.932 (19515.96)
2016	1.027 (47.0)	435.358 (19916.29)
2017	1.034 (48.8)	453.504 (20247.37)
2011–2013, average	98.3	119.3
2015–2017, average	65.9	198.9
11-13/15-17, change (%)	32.4 (-32.9)	79.6 (40.0)
IRR (CI)	0.67 (0.49–0.91)^a^	1.66 (1.32–2.09)^a^

^a^p<0,05.

**Fig 1:**
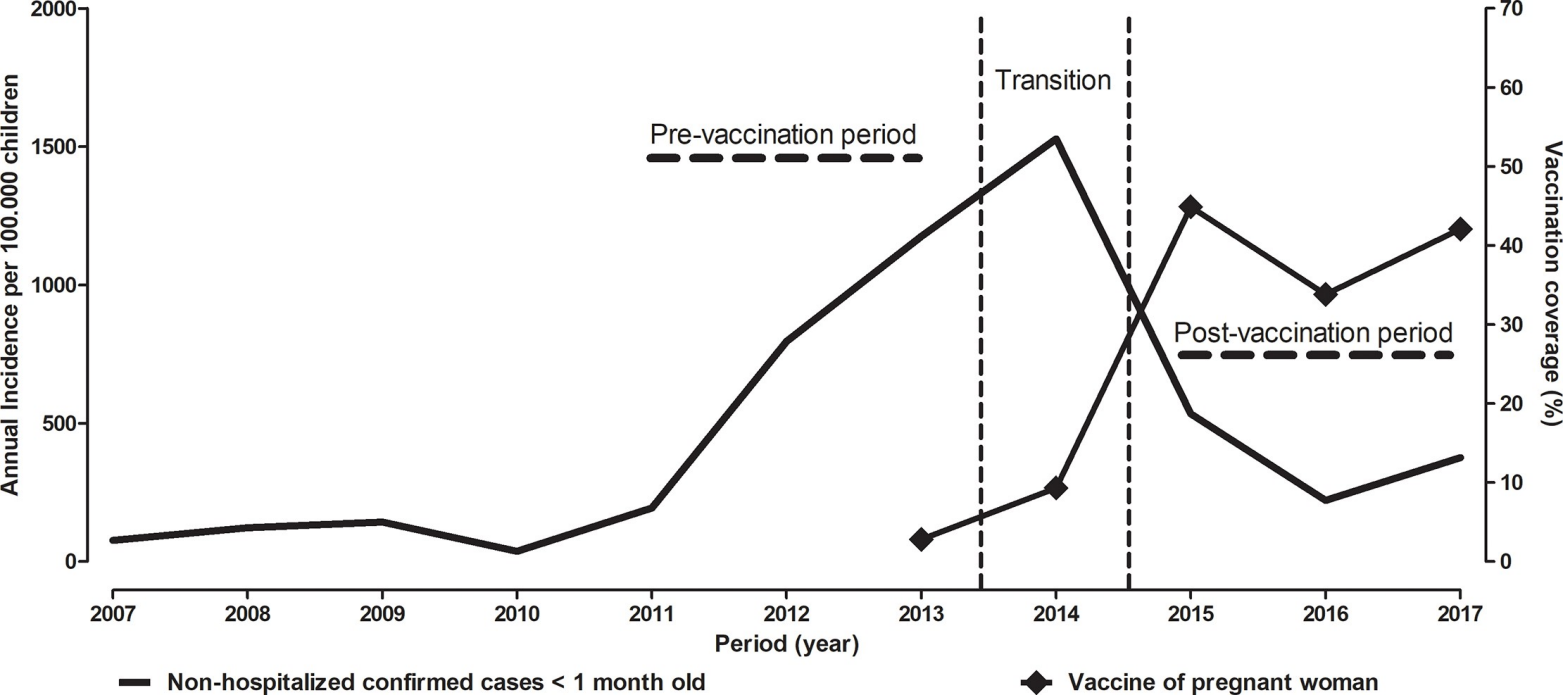
Incidence of non-hospitalized confirmed cases of pertussis in the period from 2007 to 2017 in children less than 1 month old and vaccination coverage of pregnant women in Brazil.

In relation of non-hospitalized confirmed cases, between 2007 and 2017 there were 19,344 cases in children under 1 year of age. Of these; 7,302 were confirmed by laboratory criteria, 2,111 by clinical-epidemiological criteria and 9,931 by clinical criteria. The annual distribution shows a substantial increase in the incidence of pertussis from 2011 to 2014: 194.3/100,000 in 2011, 796.7/100,000 in 2012, 1175.6/100,000 in 2013, reaching the highest number in 2014, with 1528.6/100,000 confirmed cases in children under 1 month of age. The number of cases in 2015 declined by 65% over the previous year in this age group (Fig 1).

In the pre maternal vaccination period 2011–2013, the mean annual incidence of non-hospitalized confirmed cases of pertussis in children under 1 month was 722.2 / 100,000 and in the period 2015–2017 the average was 377.3 / 100,000 [IRR 0.52 (0.46–0.59)]. In the same periods, the average incidence per year for children of one month—< 1 year aged was 64.9 / 100,000 (2011–2013) and 29.3 / 100,000 (2015–2017), representing a decrease of 54.8% [IRR 0.45 (CI 0.29–0.69)] (Table 2).

**Table 2:** Non-hospitalized confirmed cases of pertussis from 2011 to 2017 in children under 1 month and 1 month—< 1 year of age in Brazil.

Age	2011–2013	2015–2017	Absolute difference in rate^a^ (Pre and Post)	Relative difference in rate (Pre and Post)^a^	
	number of confirmed cases (number of confirmed cases per 100.000/children/year)^a^			%	IRR (CI)^b^
< 1 month					
*Average*	1366.7 (722.2)	686.3 (377.3)	-344.9	-47.7	0.52 (0.46–0.59)
*Total*	4.100 (2166.6)	2.059 (1131.9)	-1.034.7	-47.6	0.52 (0.48–0.56)
1 month—< 1 year					
*Average*	1367.0 (64.9)	584 (29.3)	-35.6	-54.8	0.45 (0.29–0.69)
*Total*	4101.0 (194.6)	1752.0 (88.0)	-106.6	-54.7	0.45 (0.35–0.58)

^a^The rate number of non-hospitalized confirmed cases per 100.000 children-years.

^b^p<0,05.

**Fig 2:**
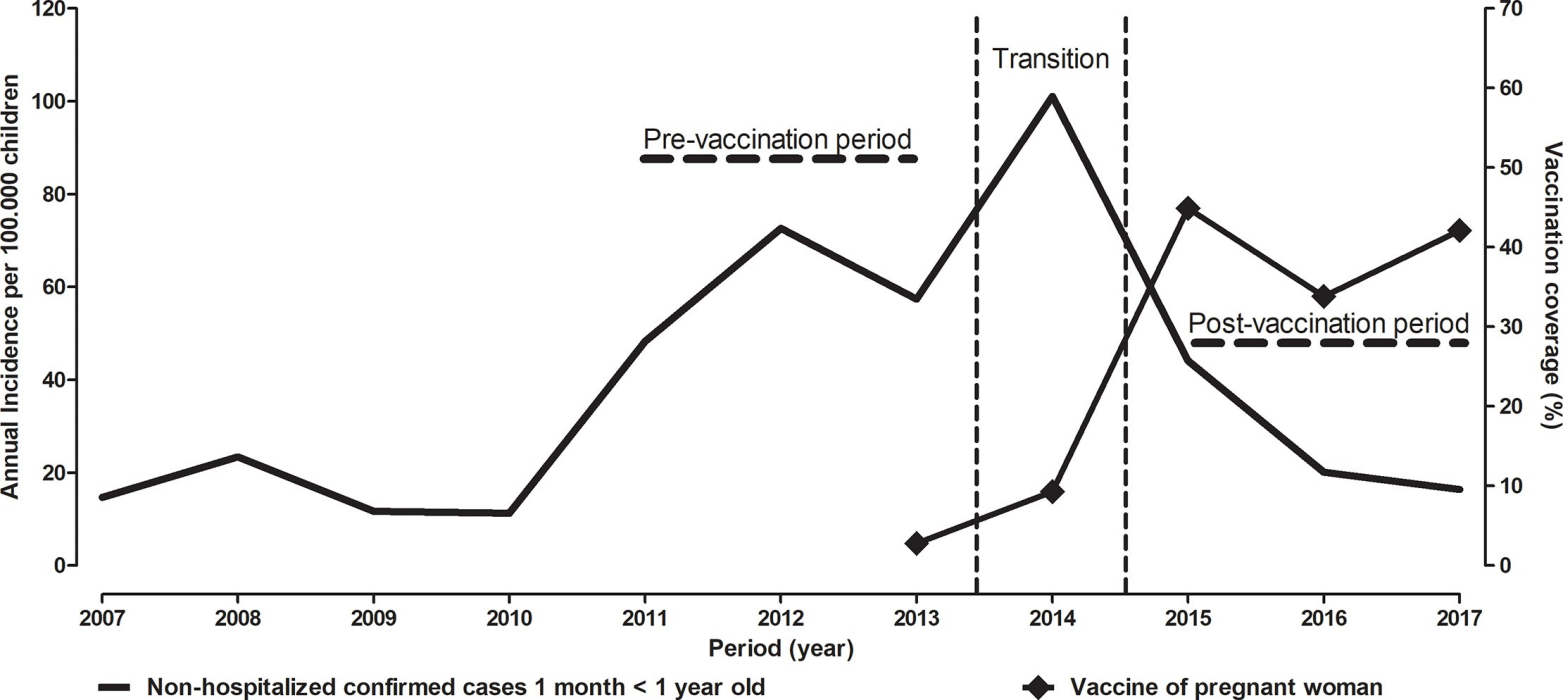
Incidence of non-hospitalized confirmed cases of pertussis from 2007 to 2017 in children with age between 1 month—< 1 year and vaccination coverage of pregnant women in Brazil.

The dTpa vaccine is offered by the National Immunization Program and is available to all pregnant women nationwide since 2013. In 2015, the program achieved approximately 44.9% coverage, coinciding with the drop in the incidence of pertussis in children with ages between 1 month—< 1 year for 48.1 / 100,000 (Fig 2).

## 4. Discussion

This is the first study demonstrating a possible impact of the vaccination of pregnant women in reducing the incidence of hospitalizations and non-hospitalized confirmed cases of pertussis in Brazil. A definitive causal relationship between vaccination during pregnancy and the following decrease of incidence in infants can´t be completely confirmed with these data, since Pertussis has a cyclic epidemiology and vaccination coverage was less than 50%. However, the reduction of incidence coincides with vaccine introduction, with a slightly greater reduction in children aged between 1 month—< 1 year.

The increase in incidence in Brazil was demonstrated in publications of 2014–2015, when DATASUS data were also used for the periods 2007–2011 and 2007–2014, respectively [9,21]. Of the confirmed cases, 23.1% occurred in children with less than three doses of the total pertussis vaccine. Of the 27 States of the Brazilian Federation, 18 did not reach 95% coverage with whole cell pertussis vaccines in 2012 [9].

Increased awareness of the disease and improved information systems may have partially contributed to a greater number of cases, since pertussis is a notifiable disease since 1975 in Brazil. In other countries, the replacement of the whole cell vaccine by acellular vaccines has been considered as a possible cause, due to its shorter immune response [22]. This view can be debated, since the disease also reappeared in countries that used whole cell vaccines, such as Brazil and Argentina [23–25].

Other possible causes of recurrence of the vaccine-related disease include the short duration of adaptive immunity following the vaccine, as well as after natural infection, which leads to the occurrence of the disease among adolescents and adults, a fact that may be a source of infection for the pediatric population [25,26].

In a previous study, the pertussis was reported in 5% of children who had received five doses of the vaccine, molecular analysis of strains identified eight clones with different antigenic variations [25]. These modifications may explain in part the decrease in vaccine efficacy, prompting the hypothesis that vaccine-induced reduced immunity may be responsible for the increased number of cases of the disease worldwide [27,28]. There is molecular evidence that circulating strains of Bordetella pertussis have changed their virulence factors, particularly the pertussis toxin and pertactin [29], although there is no data about these factors in Brazil.

Pertussis is particularly severe in children under three months of age, and the resurgence of the disease in the last decade has led to the adoption of measures that could reduce morbidity and mortality in this population. In addition to the vaccination of the contacts [30], the immunization of the pregnant woman showed an important impact by the possible transfer of maternal antibodies to the newborn [18]. The possibility of protection through the mother's vaccination was investigated in the 1930s and 1940s, but recently animal and human studies have been conducted. The dTpa vaccine used by the pregnant woman stimulates the development of anti-pertussis antibodies capable of overcoming the placental barrier, providing protection to the newborn as well as to its puerperal period, thus reducing the risk of infection [17,31].

In 2011, the immunization committee of the Centers for Disease Control and Prevention (CDC/US) recommended the use of dTpa for unvaccinated pregnant women and in 2012, the recommendation was extended to all pregnant women regardless of vaccination status and for each gestation. The use of the vaccine in different pregnancies showed no greater adverse effects when compared to vaccination in non-pregnant women. Its use was recommended between 26 and 36 weeks of gestation [8,32].

In the same period, England implemented the program of vaccination of the pregnant women for pertussis and, from confirmed cases by laboratory criteria and maternal vaccination status, the effectiveness of the vaccine could be analyzed. The study included 26,684 women who gave birth to live newborns from October 2012 to September 2013. The first nine months of 2013 showed a 78% decrease in confirmed cases [33,34]

The present study may have some limitations related to the notifications that depend on the correct diagnosis made by the attending physician and the adequate notification made by the epidemiological surveillance services, in view of this, the majority of cases had clinical confirmation. Further, we cannot select 2 months of age as the main age cut off due to the use of secondary data of a database with the cutoff at one month. This fact prevent us to assess in more detail half of the population aged less than 2 months, which is the main target of maternal vaccination. Furthermore, as mentioned previously, Pertussis may have cyclic rise on its incidence and antenatal vaccination coverage was low in Brazil. Nonetheless, reduction in incidence of all outcomes assessed coincides exactly with vaccine introduction in pregnancy, which could suggest an effect of dTpa in this reduction.

The dissemination of existing data may contribute to the adherence of prenatal services with the intention of reducing pertussis in children who are not yet vaccinated. The vaccination of the pregnant woman with dTpa from 2013 and the increase of its coverage in 2015 coincides with the reduction in the number of cases of whooping cough in children with age less than 1 year of life in 2015.

We found that the increased vaccination coverage had an impact in the incidence of pertussis, which was slightly greater in the population aged between 1 month and under 1 year with an average decrease of 54.8%, besides the IRR [0.45 (CI 0.29–0.69)]. The results may be explained by the inherited immunological response by the newborn from the mother. The reduction being greater in the older age group might reflect the previously described limitation of our cut off age of one month. This leads to the impossibility of assessment of the impact during the second month of life separately, which could be greater than in newborn, since these children had more time to be infected to Pertussis and are not old enough to be vaccinated.

Brazilian immunization program was using only the whole cellular vaccine when the incidence was increasing between 2011 and 2014. On the other side, the incidence decreased after the introduction of acellular vaccine in pregnant women in 2014. Analyzing these results, they might indicate that the replacement by acellular vaccines does not play a role in the incidence increase. However, due the cyclic nature of the epidemiology of Pertussis, an analysis of a longer period is necessary to draw any conclusion about differences between such vaccines.

The immunization of the pregnant woman seems to protect the infant through passive antibody transfer and appears to have an impact on disease prevention in infants who have not yet started the vaccination for pertussis, although further studies with a longer follow-up are necessary to confirm this association.

## Supporting information

S1 File Pertussis excel data sheet. (XLSX)
